# Coupling the environmental impacts of reactive nitrogen losses and yield responses of staple crops in China

**DOI:** 10.3389/fpls.2022.927935

**Published:** 2022-08-19

**Authors:** Ahmed I. Abdo, Daolin Sun, Yazheng Li, Jiayue Yang, Mohamed S. Metwally, Enas M. W. Abdel-Hamed, Hui Wei, Jiaen Zhang

**Affiliations:** ^1^Guangdong Laboratory for Lingnan Modern Agriculture, Guangdong Provincial Key Laboratory of Eco-circular Agriculture, South China Agricultural University, Guangzhou, China; ^2^Soil Science Department, Faculty of Agriculture, Zagazig University, Zagazig, Egypt; ^3^Department of Ecology, College of Natural Resources and Environment, South China Agricultural University, Guangzhou, China; ^4^Guangdong Engineering Technology Research Centre of Modern Eco-agriculture and Circular Agriculture, Guangzhou, China; ^5^Key Laboratory of Agro-Environment in the Tropics, Ministry of Agriculture and Rural Affairs, South China Agricultural University, Guangzhou, China

**Keywords:** acidification, global warming, aquatic eutrophication, nitrogen fertilizer, mitigation strategies, meta-analysis

## Abstract

Cropland reactive nitrogen losses (Nr) are of the greatest challenges facing sustainable agricultural intensification to meet the increases in food demand. The environmental impacts of Nr losses and their yield responses to the mitigation strategies were not completely evaluated. We assessed the environmental impacts of Nr losses in China and coupled the efficiency of mitigation actions with yield responses. Datasets about Nr losses in China were collected, converted into potentials of acidification (AP), global warming (GWP), and aquatic eutrophication (AEP), and analyzed by a meta-analysis program. Results showed that producing 1 Mg of rice grains had the highest AP (153 kg acid equiv.), while wheat had the highest GWP and AEP (74 kg CO_2_ equiv. and 0.37 kg PO_4_ equiv., respectively). Using the conventional rates (averagely, 200, 230, and 215 kg N ha^−1^) of urea as a surface application to produce 131.4, 257.2, and 212.1 Tg of wheat, maize, and rice resulted in 17–33 Tg, 7–10 Tg, and 6–87 Gg of AP, GWP, and AEP, respectively. For their balanced effect on reducing AP, GWP, and AEP while maximizing yields, inhibitors, and subsurface application could be set as the best mitigation strategies in wheat production. Inhibitors usage and biochar are strongly recommended strategies for sustainable production of maize. None of the investigated strategies had a balanced effect on rice yield and the environment, thus new mitigation technologies should be developed.

## Introduction

The grain crops (wheat, maize, and rice) are the main sources of energy and food worldwide. China is the main contributor to these crops' production by 17.9, 22.9, and 28.1%, respectively (F. A. O., 2020). However, wheat, maize, and rice represented only 11.3, 21.4, and 18.4%, respectively, of the global harvested area, which indicates an agricultural intensification of these crops in China that consumes 30% of the global produced reactive nitrogen (F. A. O., 2020). Ensuring global food security by maximizing yields while mitigating the environmental costs is a great challenge to agriculture production. This challenge is expected to grow soon because of the doubled increase in food demand by 2050 against a backdrop of growing competition for water, land, energy, labor, and climate change (Tilman et al., 2011). Reactive nitrogen (Nr) is a crucial nutrient for agricultural production and world feeding. Main source of Nr in agriculture sector is Haber–Bosch N fixation (HBNF) (Bodirsky et al., 2014). Globally, HBNF supplied ~108 Tg of Nr to agricultural uses, of which 30% is used by the main staple crops (wheat, maize, and rice) in China in 2018 (F. A. O., 2020). In parallel, the area of these crops in China represents only 9% of the world's producing area (F. A. O., 2020; Wang et al., 2020). The use efficiency of Nr decreased from 65% in 1961 to 25% in 2010, which indicated that a great amount of Nr (~270 kg N ha^−1^ yr^−1^) is being lost to the environment (Lassaletta et al., 2014). Thus, in addition to the substantial contribution of Nr in increasing food production in China, it has come at enormous environmental and economic costs (Chen et al., 2014). In monetary terms, Nr-induced pollution is assessed to cause damage to global gross domestic product by the magnitude of 0.3–3% (Sutton et al., 2013). The fates of Nr losses to the environment have been intensively assessed through emissions (NH_3_ and N_2_O gases) or leaching (${\text{NO}}_{3}^{-}$) (Bodirsky et al., 2014; Gu et al., 2015). Emissions of ammonia (NH_3_) and nitrous oxide (N_2_O) presented 7.6 and 0.2 Tg, respectively, of the total Nr (HBNF) losses that applied to the three main crops in 2018 (Abdo et al., 2020; Garba et al., 2021).

The emitted NH_3_ is the main air pollutant that contributes to acidification by forming acidic compounds (sulfate and nitrate aerosols) in the atmosphere, which is then rained out (acid rain) (Ye et al., 2011; Lindley et al., 2019). Anhydrous ammonia reacts with sulfur dioxide (SO_2_) and forms soluble (NH_4_)_2_ SO_2_ in atmospheric water vapor, which is the main source of N deposition (Vance and Peters, 2010; Liu et al., 2019). Agriculture production intensification with synthetic N fertilizers as the main input becomes a major source of global N_2_O emissions by 60% (Tian et al., 2020). Nitrous oxide (N_2_O) is a long-lived greenhouse gas and stratospheric ozone-depleting compound with an atmospheric lifetime of 116 ± 9 years (Prather et al., 2015). Aquatic eutrophication is the undesired growth of biomass production in aquatic ecosystems under higher nutrient inputs causing a shift in species ecosystems (Brentrup et al., 2004). Nitrate leaching is the main fate for diffuse N emission to aquatic ecosystems (groundwater) from soils (Wang et al., 2019b). There are no direct measures for the induced acidification (AP), global warming (GWP), and aquatic eutrophication (AEP) by Nr losses from agriculture production and none has indirectly quantified them previously. Therefore, the core idea of the current work was to address the environmental impacts of Nr losses as potentials of AP, GWP, and AEP.

A number of key mitigation strategies have been adopted to attenuate the trade-off between Nr pollution and food availability (Bodirsky et al., 2014; Chen et al., 2014). These mitigation actions included reducing the N rate, using controlled release fertilizers and inhibitors, deep placement, organic amendments, mulch, and biochar (Wang et al., 2014; Abdo et al., 2020). Although the mitigation strategies have shown the potential of reducing Nr losses and the related environmental hazards, they still have debates on increasing crop production parallelly (Huang et al., 2016; Adalibieke et al., 2021). Contrarily, setting integrated crop-soil system management actions based on a modern understanding of soil biogeochemistry and crop ecophysiology could optimize yield while reducing Nr losses (Chen et al., 2014). Additionally, the changes in crop yield in response to a mitigation strategy have been neglected in some studies that quantify the abatement costs of Nr losses (Zhang et al., 2020). Here, this study hypothesized that the direct losses of Nr applied to main crops are linked with serious and substantial environmental hazards. In addition, the mitigation strategies of these losses may be effective with one source but not another and can affect the yields that lower their applicability. Therefore, the current study aimed the quantification of AP, GWP, and AEP potentials induced by nitrogen fertilizers' application for wheat, maize, and rice production in China and the driving factors. Also, this study aimed at coupling the changes in yield and the mitigation strategies of AP, GWP, and AEP to choose the optimal actions for future planning.

## Materials and methods

Data visualization meta-analysis, statistical analysis, figures, and forest plots were implemented using R version 4.0.2 (R Core Team, 2015) and OriginLab 2021b.

### Literature review and data collection

A systematic search was used to collect a wide range of studies covering almost management practices and diverse environmental conditions to minimize bias. The publication screening was cut-off on 5 September 2021. The peer-reviewed papers were collected from Web of Science, Google Scholar, Elsevier's Scopus, and China National Knowledge Infrastructure (CNKI) due to their broad coverage and capacity to carry out intricate search strings. The keywords (ammonia emission or volatilization, nitrous oxide emission, nitrate leaching, environmental impact assessment, nitrogen fertilizers, acidification, global warming, and aquatic eutrophication) were used to screen databases. More than 600 papers were screened and 204 papers were selected (Data S1) with 1,893 datasets. These datasets were assessed for their relevance to the current study by the inclusion of the following criteria:

- Experimental details include study location, type, setup year, replicates number, crop type (maize, wheat, and rice), climatic conditions (air temperature and precipitation), and soil parameters [pH, soil organic matter content (SOM) clay content, and total nitrogen content (STN)]. Field and lysimeters studies only were included in the meta-analysis, while watershed-scale or modeling calculations and lab-based studies were excluded (Huddell et al., 2020).
- Agronomic practices such as fertilizer type (urea, other mineral N fertilizers, organic amendments, and slow released fertilizers), rate and application depth, irrigation and amendments including biochar, inhibitors (urease and nitrification inhibitors, mainly N-(n-butyl) thiophosphoric triamide dicyandiamide), and mulching.
- Accumulative seasonal Nr losses (NH_3_, N_2_O, and ${\text{NO}}_{3}^{-}$) as a percentage or kg ha^−1^, grain yield (Mg ha^−1^), standard deviation (STD), and standard error (SE).

**Figure 1:**
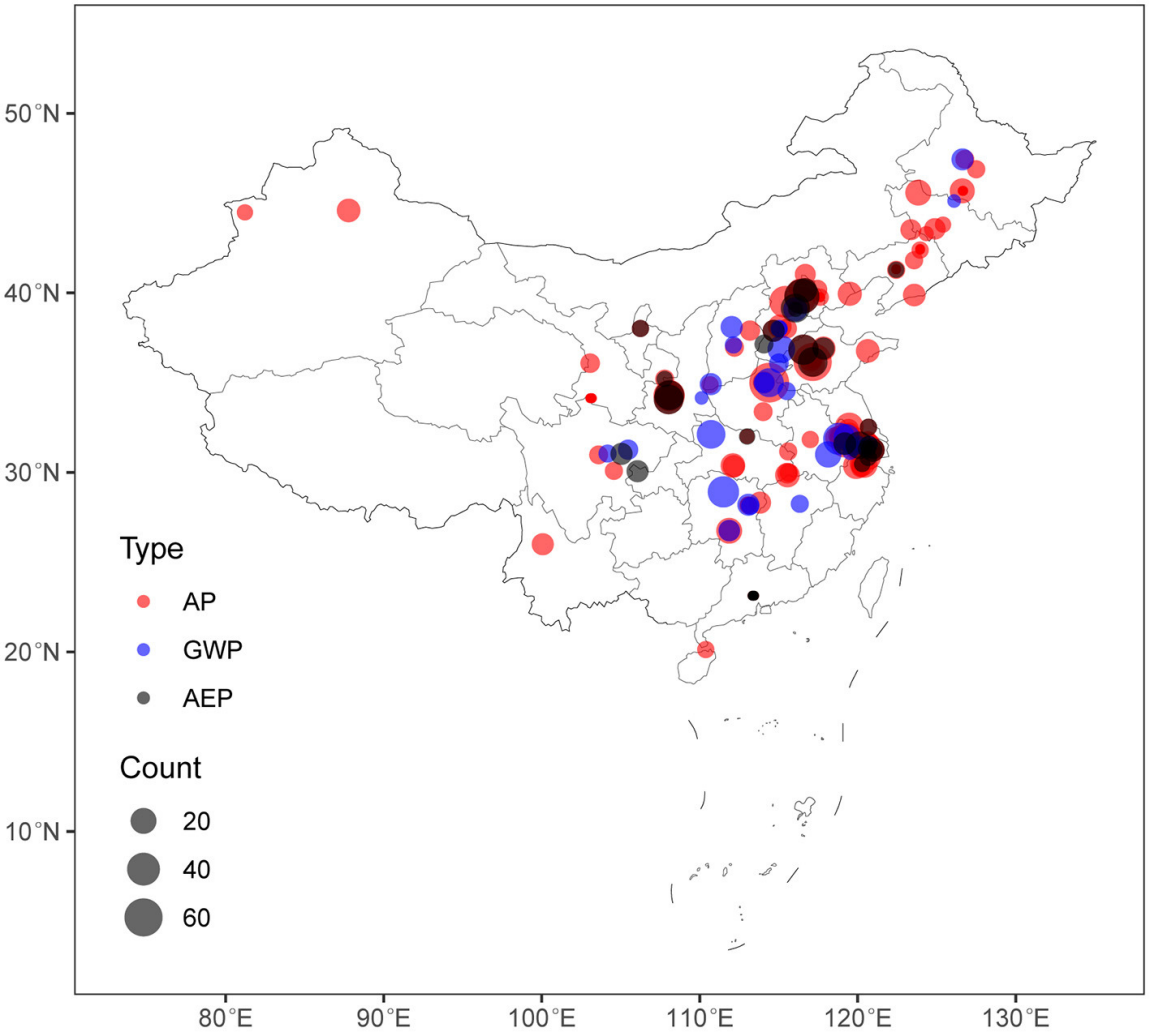
Map of the dataset sites collected in this study. The symbol color indicates the type of environmental impact and the size indicates observations number within each site (*n* = 1,030 sites for AP, *n* = 398 sites for GWP, *n* = 365 sites for AEP). AP is air acidification potential (Acid equiv. Mg^−1^ grains), GWP is the global warming potential (kg CO_2_ equiv. Mg^−1^ grains) and AEP is the aquatic eutrophication potential (kg PO_4_ equiv. Mg^−1^ grains).

Data from the papers that fit our study criteria were extracted and input into the Microsoft excel 2013 package. The figures were digitized using the software Plot Digitizer to extract the numerical data that are not available in paper text (Huwaldt, 2015). Next, the missing data were completed. The sites of observations (Figure 1) were drawn using the “tmap” package (Tennekes, 2018). Almost wheat and maize studies came from Northern to Middle China and rice studies from Southern China with 60, 21, and 19% of tested NH_3_, N_2_O, and ${\text{NO}}_{3}^{-}$. The trial site, year, crop type, and growth season period were used to extract the missing climatic data from China Meteorological Data Service Center (CMDC). Then, daily observations of temperature and precipitation were summed during the crop growth season to calculate the seasonal accumulation. The irrigation amount (mm) during growth season was summed with the seasonal precipitation (mm) to calculate the total water input as water load (mm). Missing data on soil properties (pH, SOM, STN, and clay) were collected using databases and other papers that have been carried out at the same experimental site. Missed STDs were imputed using SE, coefficient of variance (CV), p-values, confidence interval (CI), and t-values using the meta-analysis software (Borenstein et al., 2009; Nkebiwe et al., 2016), using the following equations (Equations 1–5):


(1) $$\begin{array}{l} V_{D}\;=\;\frac{n_{1}\;+\;n_{2}}{n_{1}n_{2}}\;{\times \;S}_{pooled}^{2} \end{array}$$

(2) $$\begin{array}{l} W\;=\;\frac{1}{V_{D}\;+\;\tau^{2}} \end{array}$$

(3) $$\begin{array}{l} S_{pooled}\;=\;\sqrt{\frac{\left(n_{1}\;-\;1\right)S_{1}^{2}\;+\;\left(n_{2}\;-\;1\right)S_{2}^{2}}{n_{1}\;+\;n_{2}\;-\;2}} \end{array}$$

(4) $$\begin{array}{l} SE_{D}\;=\;\sqrt{V_{D}} \end{array}$$

(5) $$\begin{array}{l} \bar{ES}\;=\;\frac{\sum_{i=1}^{k}W_{i}ES_{i}}{\sum_{i=1}^{k}W_{i}} \end{array}$$

Where *n*_1_ and *n*_2_ are sample sizes of treatments and control, $S_{pooled}^{2}$ is the squared standard deviation of pooled effect size ($\bar{ES}$), *SE*_*D*_ is the standard error, *V*_*D*_ is the variance of difference in means, *W* is the weight, *ES* is the effect size for each variable, τ is variance between groups, and *k* is the number of effect sizes within the group.

### Calculating the potentials of acidification, global warming, and aquatic eutrophication and grouping

First, the yield scaled Nr losses were calculated using the following equation [Equation 6 (Wang et al., 2014)]:


(6) $$\begin{array}{l} \text{Nr losses }\left(\text{kg N }Mg^{-1}\right)=\frac{Nr_{i}}{GY} \end{array}$$

where *Nr*_*i*_ is the nitrogen losses (kg N ha^−1^) via *i* which represents NH_3_, N_2_O, or ${\text{NO}}_{3}^{-}$ and GY is the grain yield (Mg ha^−1^). Then, Nr losses were converted into AP [Equation 7 (Lindley et al., 2019)], GWP [Equation 8 (IPPC, 2006; Cui et al., 2013)], and AEP [Equation 9 (Seppäl et al., 2004)]:


(7) $$\begin{array}{l} AP\;=\;Nr_{NH_{3}}\;\times \;58.75 \end{array}$$

(8) $$\begin{array}{l} GWP\;=\;Nr_{N_{2}O}\;\times \;298 \end{array}$$

(9) $$\begin{array}{l} AEP\;=\;Nr_{NO_{3}^{-}}\;\times \;0.1 \end{array}$$

where AP is the acidification potential (Acid equiv. Mg^−1^ grains), GWP is the global warming potential (kg CO_2_ equiv. Mg^−1^ grains), and AEP is the aquatic eutrophication potential (kg PO_4_ equiv. Mg^−1^ grains). *Nr*_*NH*_3__, *Nr*_*N*_2_*O*_, and $Nr_{NO_{3}^{-}}$ are the Nr losses through different sources (*kg N Mg*^−1^) and the equivalency factors for AP, GWP, and AEP are 58.75, 298, and 0.1, respectively.

After data completion and arrangement, datasets were grouped into three main groups (wheat, maize, and rice) including every three subgroups, which represent AP, GWP, and AEP.

Under each subgroup, datasets were classified based on N source into four categories including urea, other synthetic fertilizers (OCF), improved urea (IU, slow released fertilizers), and organic sources (OA). According to the previous studies, the conventional N rates ranged between 150 and 250, 200 and 260, and 170 and 260 kg N ha^−1^ for wheat, maize, and rice, respectively (Zhang et al., 2013; Chen et al., 2014; Tingyu et al., 2020). Therefore, the observations were categorized based on N rates into three groups representing 0 < R1 < 150 (the reduced N rate strategy), 150 < R2 < 250 (the conventional rate), and R3 > 250 (the excessive use scenario). The collected observations showed biochar, mulch, and inhibitors as common amendments that have been used to mitigate Nr losses. Thus, the datasets were grouped into those three amendments categories under the conventional N rate of urea as a common N fertilizer source in China (Abdo et al., 2020). Another important strategy for controlling Nr losses is the subsurface application; here, we divided the datasets into two groups (surface and subsurface applications) under R2 of urea.

### Quality control of data

The quality control for raw and calculated data was implemented, wherein the normality test was done using the Shapiro–Wilk normality test at *p* < 0.05. The average SD/mean ratio was estimated using the bootstrap method to adjust the normality of non-normal distributed data (Jian et al., 2020). Publication bias at *p* > 0.05 was tested graphically using funnel plot “funnel” and the fail-safe number “fsn” functions in “metafor” (Viechtbauer, 2010). Heterogeneity was tested at *p* ≤ 0.05 using the “r2_ml” function in the R package “orchard.” The test confirmed that variations among pairwise comparisons were greater than the sampling error as all estimated effect sizes were located within the pooled ES and 95% confidence interval (CI) limits.

### Averages of acidification, warming, and eutrophication potentials and driving factors

The meta-analysis was implemented after quality control for each pairwise comparison to calculate the effect sizes and pooled effect size of each group using the “escalc” function, R package METAFOR (Viechtbauer, 2010). Here, the effect sizes represent the difference in means between the treatment and control of each observation (Data S1), while pooled effect size was used for comparison between groups by combining one effect size of all datasets within the same group (Pathak et al., 2020). Random effect models were used to estimate the pooled effect sizes for their stability with a small standard error-based confidence interval (Rosenberg et al., 1997). First, we implemented the meta-analysis to calculate the overall means of AP, GWP, and AEP (kg Mg^−1^ grains) for each crop under R2 of urea.

The effects of environmental drivers (air temperature, water load, soil pH, SOM, STN, and clay content) on AP, GWP, and AEP were tested using a mixed-effects meta-regression model in the “glmulti” package in R. Each driver was expressed as the sum of Akaike weights in the regression model to select the important factors across models, wherein 0.8 was set as the cutoff between unimportant and important predictors (Terrer et al., 2016). The important drivers had a significant effect at *p* < 0.05.

### Coupling the effects of mitigation strategies with yield responses

First, we performed a meta-analysis again incorporating mitigation strategies as moderators, given that AP, GWP, and AEP (kg Mg^−1^ grains) under urea, R2, surface application, and no amendments were the baselines to evaluate the effects of these strategies (Supplementary Figures 1A–D). In parallel, we assessed the effect of these strategies' actions on the grain yield of each crop through the meta-analysis to couple changes in crop production with reductions in AP, GWP, and AEP under each strategy (Adalibieke et al., 2021).

Second, we used the differences in means of baselines to calculate the total of AP (Tg), GWP (Tg), and AEP (Gg) in China (Equations 10, 11, and 12) as the sum of all Provinces (Supplementary Table 1):


(10) $$\begin{array}{l} AP_{total}\;=\;\frac{\sum_{i=1}^{k}\;AP_{i}\times GY_{i}}{10^{9}} \end{array}$$

(11) $$\begin{array}{l} GWP_{total}\;=\;\frac{\sum_{i=1}^{k}GWP_{i}\times GY_{i}}{10^{9}} \end{array}$$

(12) $$\begin{array}{l} AEP_{total}\;=\;\frac{\sum_{i=1}^{k}\;AEP_{i}\times GY_{i}}{10^{6}} \end{array}$$

where *AP*, *GWP*, and *AEP* are the differences in means (kg Mg^−1^ grains) calculated by the meta-analysis. GY is the grain yield (Mg) of each crop in a province, *i* is the province number, *k* is the total number of provinces included in the calculations (31 Provinces), and 10^9^ and 10^6^ were used to convert kg to Tg and Gg, respectively.

Third, we calculated the effect of each strategy on yield and AP, GWP, and AEP as ratios of the baseline means (Equation 13):


(13) $$\begin{array}{l} Srategy\;effect\;\left(\%\right)\;=\;\frac{Db_{i}\;-\;Ds_{i}}{Db_{i}}\;\times \;100 \end{array}$$

where *D*_*b*_ and *Ds* are differences in means of baseline and a mitigation strategy, respectively. *i* refers to grain yield (Mg), or AP, GWP, and AEP (kg equiv. Mg^−1^). Positive values mean decreases down the baseline after implementing a mitigation strategy, while negative values mean that this strategy had an increasing effect over the baseline.

Fourth, we used these ratios to calculate the effect of each strategy on total yield (Tg) and AP, GWP, and AEP (Tg) in China (Equation 14):


(14) $$\begin{array}{l} Strategy\;effect\;\left(Tg\right)\;=\;Tb_{i}\\ -\;\left(Tb_{i}\;\times \;\frac{Strategy\;effect_{i}\;\left(\%\right)}{100}\right) \end{array}$$

where *Tb* is the total yield (Tg) or AP, GWP, and AEP (Tg) under baselines and *i* refers to the strategy type which included two N rates (R1 and R3), three fertilizer types (OCF, IU, and OA), subsurface application, and three amendments (mulch, biochar, and inhibitors).

## Results

### Overall environmental impacts of reactive N losses

**Figure 2:**
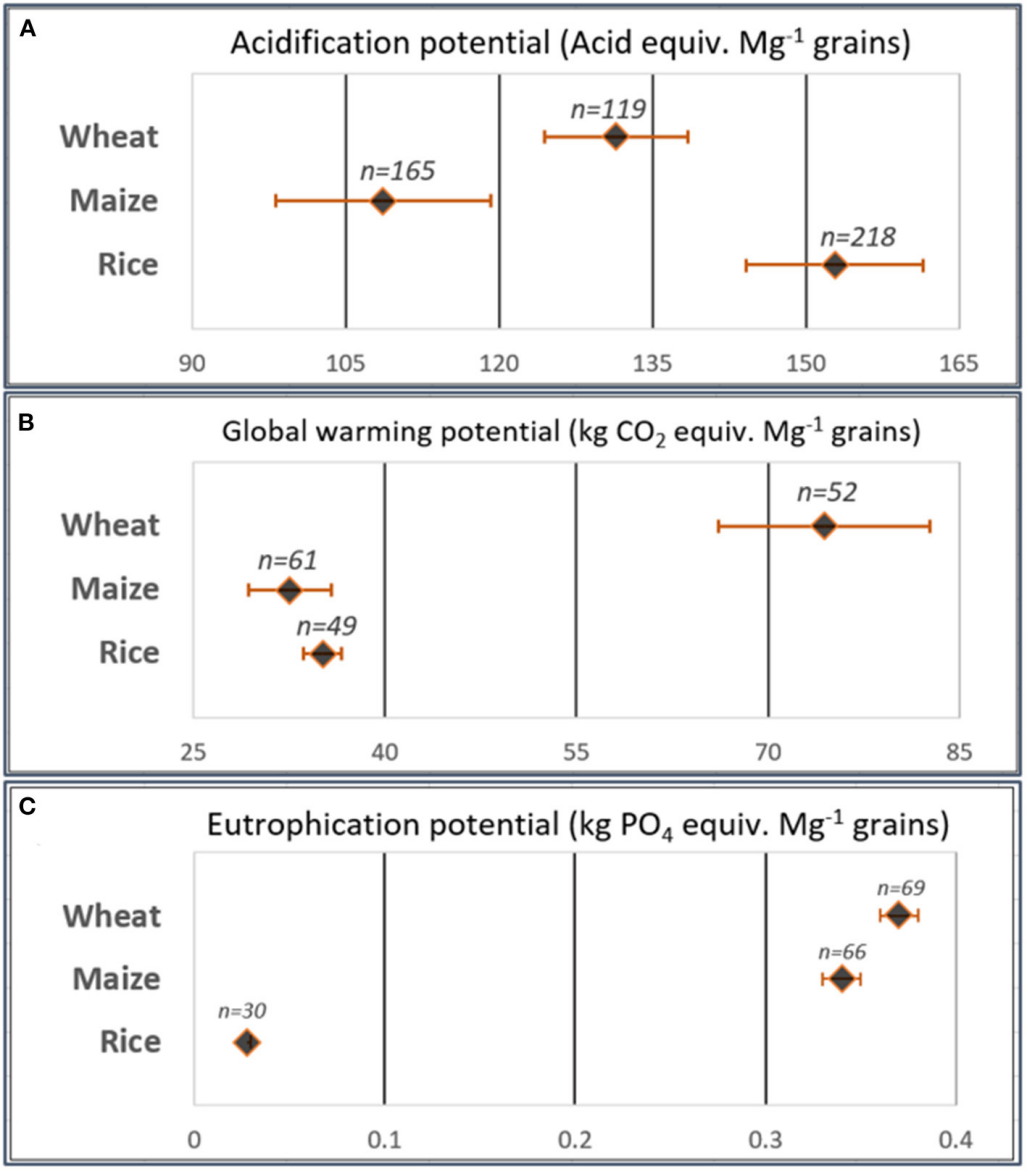
**(A)** The overall acidification potential (Acid equiv. Mg^−1^ grains), **(B)** global warming potential (kg CO_2_ equiv. Mg^−1^ grains) and **(C)** aquatic eutrophication potential (kg PO_4_ equiv. Mg^−1^ grains) resulted from the meta-analysis program. These are the pooled effect sizes of each group calculated using the observations received urea fertilizers at conventional rates (between 150–250, 200–260, and 170–260 kg N ha^−1^ for wheat, maize and rice, respectively) and applied as surface application without any amendments.

Producing 1 Mg grains of rice had the highest potential of acidification (153 kg acid equiv.) followed by wheat (131 kg acid equiv.), while maize had the lowest AP by 109 kg acid equiv. Mg^−1^ grains (Figure 2A). Also, maize had the lowest GWP (33 kg CO_2_ equiv. Mg^−1^ grains) followed by rice (35 kg CO_2_ equiv. Mg^−1^ grains), while wheat recorded the highest GWP (74 kg CO_2_ equiv. Mg^−1^ grains) (Figure 2B). Producing 1 Mg grains of wheat and maize had the highest AEP (0.37 and 0.34 kg PO_4_ equiv., respectively), while rice recorded the lowest AEP (0.03 kg PO_4_ equiv. Mg^−1^ grains) (Figure 2C).

### Driving factors of Nr-induced environmental effects

**Figure 3:**
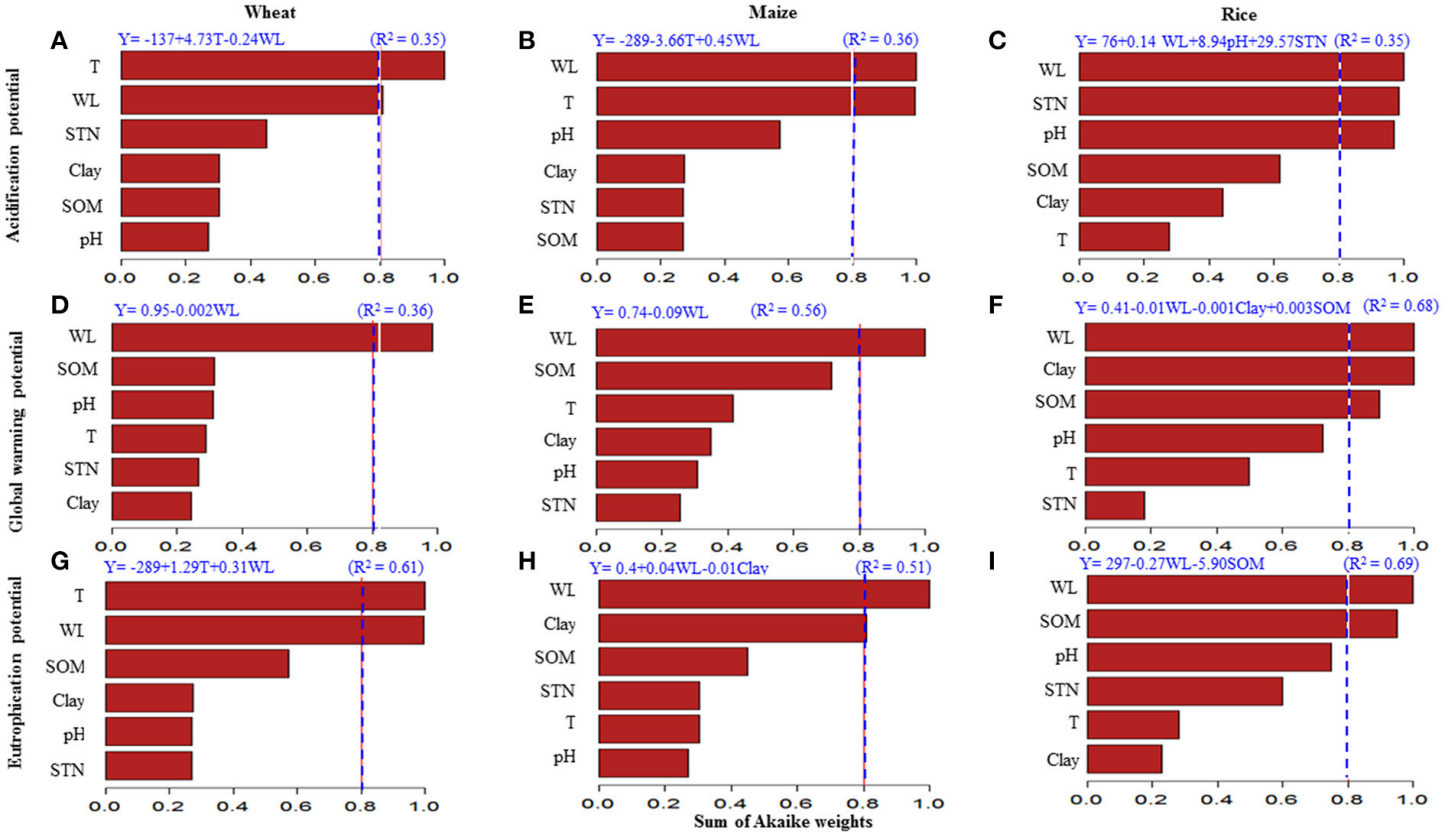
**(A–I)** Model-averaged importance of the drivers controlling the changes in overall AP, GWP, and AEP. The importance was calculated based on the sum of Akaike weights by the model selection using AICc. To differentiate between the unimportant and important drivers, we set 0.8 as the cutoff (dashed line). T is the overall temperatures during the growth season (°C). WL is the water load (mm) during season calculated by summation of precipitation (mm) and irrigation (mm). SOM is the soil organic matter content (g kg^−1^). STN is the soil total nitrogen (g kg^−1^) and clay is the soil clay content (g kg^−1^). The upper equations are mixed-effects meta-regression models at *p* < 0.05 and *R*^2^ is the relation strength.

Water load and temperature were the main drivers affecting AP in wheat and maize (Figures 3A,B) with a moderately weak relation (*R*^2^= 0.35 and 0.36, respectively), while in rice, the dominant drivers were water load, STN, and pH (*R*^2^= 0.35) (Figure 3C). The multiple-regression equations revealed that water load increased AP in maize and rice, while decreased AP in wheat (Figures 3A–C). Air temperature increased AP in wheat, while decreased AP in maize. Soil pH and STN increased AP in rice.

Water load reduced GWP in wheat and maize as the sole environmental driver with moderate relation (*R*^2^= 0.36 and 0.56, respectively) (Figures 3D,E). Besides the negative effect of water load, soil clay content and SOM were the main environmental drivers affecting the GWP in rice with a moderately strong correlation (*R*^2^= 0.68) (Figure 3F). Clay content decreased GWP, while SOM increased it. The water load was a dominant driver of GWP in rice with a negative effect.

Air temperature and water load were the dominant factors affecting AEP in wheat positively with moderate strong relation (*R*^2^= 0.61) (Figure 3G). The regression model showed an increasing effect of water load and decreasing effect of clay content on AEP in maize (Figure 3H). Water load followed by SOM were the main drivers of AEP changes in rice with a reduction effect and strong relation (*R*^2^= 0.69) (Figure 3I).

### Coupling environmental impacts and crop yield under the mitigation actions

#### Wheat

**Figure 4:**
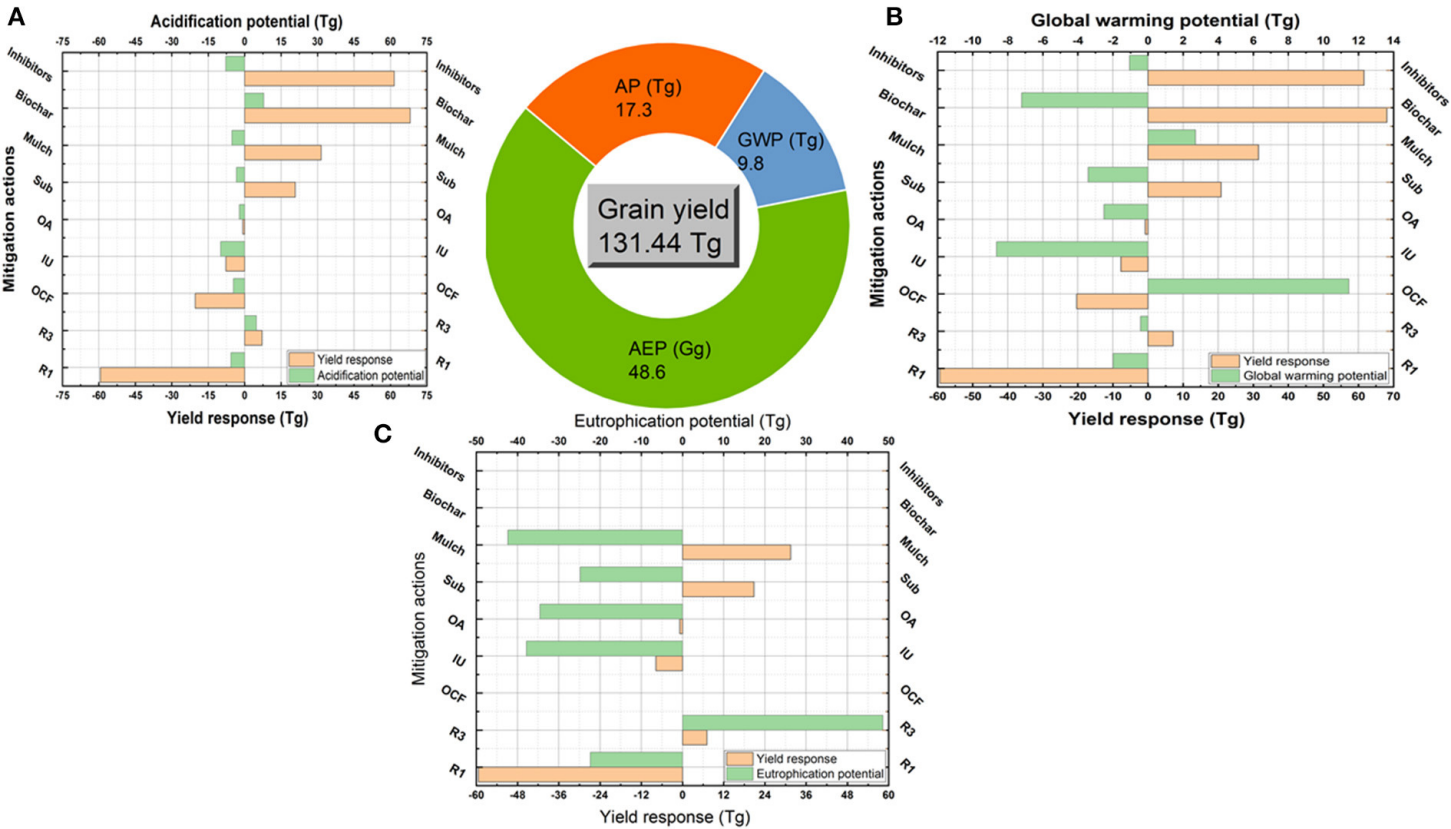
Coupled yield production and environmental impacts in wheat crop. Pie chart refers to the total produced grains (Tg) under the conventional practices (using urea fertilizer at conventional rates with surface application and no amendments) and the total coupled AP (Tg Acid equiv.), GWP (Tg CO_2_ equiv.) and AEP (Gg PO_4_ equiv.). Column figures refer to the coupled changes in yield and AP **(A)**, GWP **(B)**, and AEP **(C)** as responded to each strategy. These strategies are reduced N rate (R1), increased N rate scenario (R3), other synthetic fertilizers (OCF), improved urea (IU, slow released fertilizers), organic sources (OA), subsurface application (Sub) and uses of mulch, biochar and inhibitors.

Producing 131.4 Tg of wheat grains in 2018 was coupled with 17.3 Tg acid equiv., 9.8 Tg CO_2_ equiv., and 48.6 Gg PO_4_ equiv. of AP, GWP, and AEP, respectively (Pie chart, Figure 4). Decreasing N application rate lowered the AP, GWP, and AEP by 5.7 Tg, 2.0 Tg, and 22.4 Gg, respectively, but was coupled with the minimum yield (59.4 Tg) (Figures 4A–C). In contrast, the increased N rate scenario resulted in the highest AP (4.9 Tg) and AEP (48.6 Tg) coupled with yield increment (7.2 Tg) but not much like the yield reductions under low N rates. Replacing urea with other mineral fertilizers reduced AP by 4.6 Tg and maximized GWP by 11.5 Tg, while was coupled with yield reduction by 20.3 Tg. The improved urea strategy resulted in the minimum AP, GWP, and AEP (9.9, 8.6, and 37.8 Gg, respectively), and also reduced the grain yield by 7.7 Tg. Partial replacement of urea by organic amendments reduced AP, GWP, and AEP and decreased yield slightly. Mulch strategy maximized yield by 31.5 Tg and reduced AP and AEP significantly, but increased GWP by 2.7 Tg. Using inhibitors and subsurface applications were the best strategies to reduce AP, GWP, and AEP notably and at the same time maximized the yield by 61.6 and 20.8 Tg, respectively.

#### Maize

**Figure 5:**
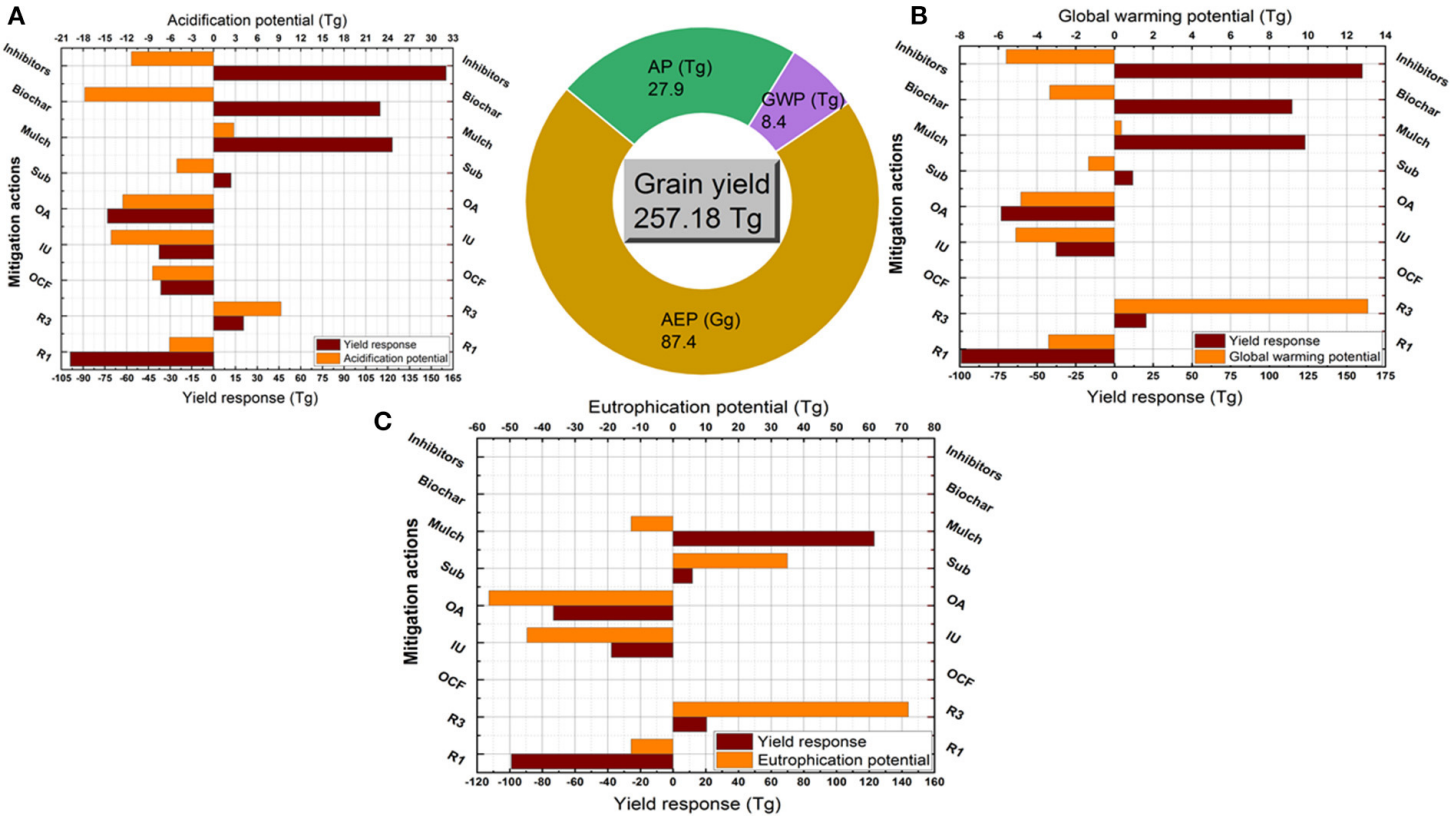
Coupled yield production and environmental impacts in maize crop. Pie chart refers to the total produced grains (Tg) under the conventional practices (using urea fertilizer at conventional rates with surface application and no amendments) and the total coupled AP (Tg Acid equiv.), GWP (Tg CO_2_ equiv.), and AEP (Gg PO_4_ equiv.). Column figures refer to the coupled changes in yield and AP **(A)**, GWP **(B)**, and AEP **(C)** as responded to each strategy. These strategies are reduced N rate (R1), increased N rate scenario (R3), other synthetic fertilizers (OCF), improved urea (IU, slow released fertilizers), organic sources (OA), subsurface application (Sub) and uses of mulch, biochar and inhibitors.

Producing 257.2 Tg of maize grains in 2018 was coupled with 27.9 Tg acid equiv., 8.4 Tg CO_2_ equiv., and 87.4 Gg PO_4_ equiv. of AP, GWP, and AEP, respectively (Pie chart, Figure 5). Reducing urea rate or replacing by other mineral fertilizers and improving urea or organic amendments reduced AP by 6 to 14 Tg, GWP by 3 to 5 Tg, and AEP by 12 to 56 Gg (Fig. 5a, b, and c), but strongly reduced the yield by 99, 36.5, 37.7, and 73.1 Tg, respectively. Contrarily, increasing the N rate raised yield by 20.5 Tg, but increased AP by 9.3 Tg, GWP by 13.1 Tg, and AEP by 72.0 Gg. Using inhibitors and biochar were the best actions to strongly mitigate AP and GWP and coupled with maximizing yield by 160.1 and 114.7 Tg. Mulch strategy maximized yield and reduced AEP, but increased AP and GWP. Subsurface action increased yield and decreased AP and GWP, but increased AEP.

#### Rice

**Figure 6:**
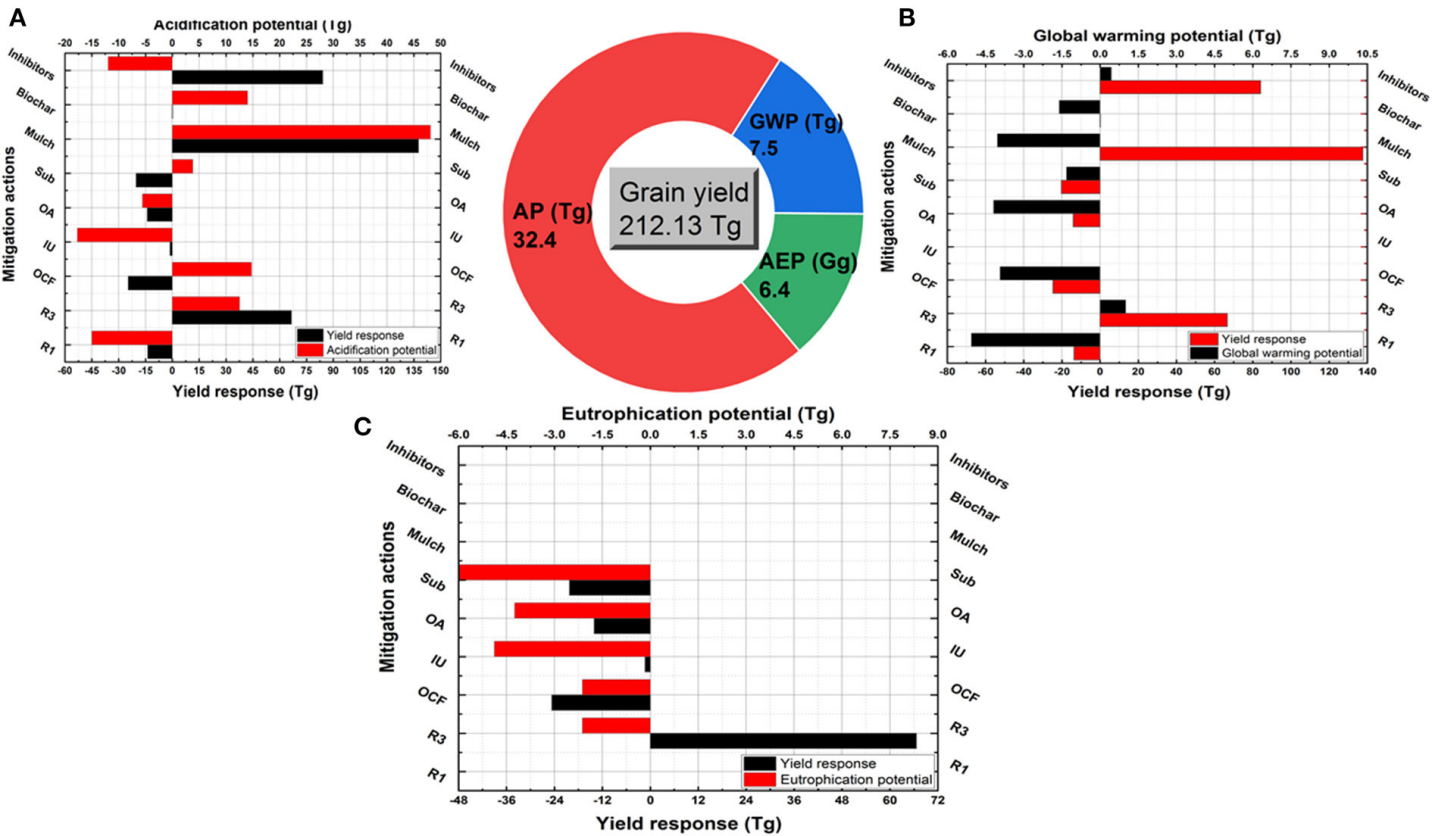
Coupled yield production and environmental impacts in rice crop. Pie chart refers to the total produced grains (Tg) under the conventional practices (using urea fertilizer at conventional rates with surface application and no amendments) and the total coupled AP (Tg Acid equiv.), GWP (Tg CO_2_ equiv.) and AEP (Gg PO_4_ equiv.). Column figures refer to the coupled changes in yield and AP **(A)**, GWP **(B)**, and AEP **(C)** as responded to each strategy. These strategies are reduced N rate (R1), increased N rate scenario (R3), other synthetic fertilizers (OCF), improved urea (IU, slow released fertilizers), organic sources (OA), subsurface application (Sub) and uses of mulch, biochar and inhibitors.

Producing 212.1 Tg of rice grains in 2018 was coupled with 32.4 Tg acid equiv., 7.5 Tg CO_2_ equiv., and 6.4 Gg PO_4_ equiv. of AP, GWP, and AEP, respectively (Pie chart, Figure 6). None of the investigated strategies achieved both yield optimization and all environmental hazards mitigation in rice (Figures 6A–C). Reduced urea rate or replaced by improved urea or organic amendments reduced AP by 1–14 Tg, GWP by 5–18 Tg, and AEP by 4–5 Gg. Using other N fertilizers and subsurface application reduced GWP and AEP, but increased AP and decreased yield. Contrarily, inhibitors decreased AP but increased GWP and yield. Mulch maximized yield by 137.7 Tg and biochar sustained yield by a slight increase (0.2 Tg), both of them reduced GWP notably but increased AP strongly (14–48 Tg).

## Discussion

### Potentials of acidification, global warming, eutrophication, and the driving factors

Results demonstrated higher potentials of acidification by Nr losses from rice fields than wheat and then maize (Figure 2A). Emitted NH_3_ is the basis of calculating AP in the current study, it has been reported that crop type influenced the magnitude of NH_3_ volatilization with the highest intensity in rice as compared with wheat and maize (Huang et al., 2016). The main area of rice production is Southern China which is a subtropical region with high precipitation. High pH in rice paddies due to diurnal algal photosynthesis and the limited ability to buffer solution ${\text{NH}}_{4}^{+}$ under increased water load (flooding) caused higher AP in rice fields (Sommer et al., 2004). Additionally, N fertilizer is surface broadcast on water surface in a rice paddy as conventional pre-planting practice, which induced higher potentials of NH_3_ volatilization (Peng et al., 2010). The wheat growth period is two times more than that of rice or maize; however, wheat AP is less than that of rice as around 4 months of wheat growth season are under extremely cold conditions. Wheat is the common crop in cold arid regions in North China, which indicated lesser AP than rice. This is related to the increasing effect of temperature on wheat AP (Figure 3A) and the results by Chen et al. (2014) about NH_3_ emissions from the three crops.

Producing a unit of wheat grains contributed to global warming higher than rice and maize (Figure 2B). This agrees well with findings by Chen et al. (2014) and Garba et al. (2021) when neglecting the CH_4_-induced GHG. Lower GHG by rice than wheat is mainly due to the lower nitrification rate under paddy conditions. Aerated conditions are the most favorable for the nitrification process, which is the typical pathway for N_2_O production—the main component of N fertilizers-induced GHG—under wheat and maize upland fields (Barnard et al., 2005). This is related to the negative relation between GWP and water load in the three crops (Figures 3D–F). Higher SOM content increased the GWP due to increasing the microbial activity, nitrification process, and N_2_O production (Das and Adhya, 2014). This explained the positive relation between rice GWP and SOM (Figure 3F). The regression model showed a negative relation between rice GWP and clay content (Figure 3F) due to higher absorbency of ${\text{NH}}_{4}^{+}$ and lesser N_2_O emissions under increased soil colloidal contents (Lin et al., 2012). Maize is a summer crop affordable to excessive precipitation during the growth season, which decreased the GWP lower than wheat (Figure 2B) owing to the negative relation between water load and maize GWP (Figure 3E). Moreover, maize induced lesser GWP than paddy rice, which refers to another main loss pathway represented by nitrate leaching as AEP in the current study (Figure 2C).

This study showed that AEP was similar in wheat and maize, which was 12 times higher than rice AEP (Figure 2C), agreeing with the results obtained by Zhou and Butterbach-Bahl (2014) and Yang et al. (2018). The main driving factor controlling AEP in the three crops was water load, wherein intensive water load events were more likely to drive the greater potential of nitrate leaching in wheat and maize (Figures 3G,H) (Liang et al., 2011). Wheat is planted in Northern China (7–14°C and 20–1,000 mm annual precipitation) and rice in Southern China (15–24°C and 1,000–2,000 mm annual precipitation), while maize is planted across all regions (Wang et al., 2019a). These variations in production areas derived the changes in wheat AEP as affected by temperature and water load, while maize and rice were affected mainly by water load only (Figures 3G–I). Also, these variations in maize areas across China with different soil textures derived the reduction effect of clay content on maize AEP (Figure 3H). Nitrate leaching in clay soils with high nutrient preserving capability is less than that in sandy soils (Lu et al., 2019). The effect of clay content was not present on AEP in wheat as it is planted in Northern areas with sandy to loamy textures or rice AEP as it is planted in Southern areas with a clay texture. Water load decreased rice AEP due to reducing the nitrification rate under waterlogging conditions in paddy fields (Das and Adhya, 2014). The negative relation between rice AEP and SOM (Figure 3I) is in the same consent as findings by Malcolm et al. (2019) who reported lesser nitrate leaching under higher SOM contents due to improving nutrient-holding capacity.

### Yield-cost effective mitigation strategies

Maximizing yields while reducing environmental hazards in sustainable intensification of wheat, maize, and rice systems is a great challenge, especially under intensive nitrogen fertilizers application. Using the conventional N rates (averagely, 200, 230, and 215 kg N ha^−1^) resulted in producing 131.4, 257.2, and 212.1 Tg of wheat, rice, and maize grains, respectively (Figures 4, 5). These yields were coupled with an environmentally adverse load of AP, GWP, and AEP by amounts ranging between 17 and 33 Tg, 7 and 10 Tg, and 6 and 87 Gg, respectively. That ensures the urgent need to mitigate the Nr losses radically. Reducing the N rate was an effective strategy to mitigate AP, GWP, and AEP in the three crops, but it decreased yield notably. That eliminates the application of this strategy, especially in future plans under a significant increase in population and food demand. Contrarily, the excessive use of N fertilizers indicated significant increases in yields coupled with great increases in AP, GWP, and AEP. Simply, farmers are willing to increase the N rate as it increases yields but do not bear the risk of reducing the N rate as it would reduce yield production (Hvistendahl, 2010). Serious restrictions must be taken to prevent excessive use of N fertilizers, if not, China's environment will continue to deteriorate (Huang and Sass, 2010).

Managing the N rate applied to cereal crops has to be accompanied by other strategies to increase nitrogen use efficiency and optimize yield production. We assessed the most common suggested strategies for mitigating Nr losses and subsequently their environmental impacts. We found that subsurface application and using inhibitors could be set as the best options for mitigating the environmental impacts of Nr losses under wheat production. The two strategies reduced AP, GWP, and AEP in wheat coupled with significant increases in yield production. Both of them reduced Nr losses by decreasing the urease activity and ${\text{NH}}_{4}^{+}$ contents in paddy floodwater and surface soils and enhancing the ${\text{NH}}_{4}^{+}$ immobilization (Liu et al., 2015). Inhibitors usage was also the best strategy for mitigating AP, GWP, and AEP in maize and maximizing yield, while the subsurface application was an effective strategy with yield, AP and GWP but not AEP. This refers to more nitrate leaching with the deep placement of N fertilizers under maize field conditions with excessive water load (Zhou and Butterbach-Bahl, 2014). Moreover, biochar was also an effective strategy to reduce the environmental impacts of maize production coupled with yield maximization. This result is consistent with findings by Huang et al. (2016) about ammonia emissions, Zhou and Butterbach-Bahl (2014) about nitrate leaching, and He et al. (2018) about nitrous oxide emission. None of all investigated strategies achieved balanced reductions in AP, GWP, and AEP and increases in rice yield, thus new mitigation technologies should be developed.

Interestingly, the current study indicated different efficiencies of the investigated strategies on yield and the environment among the three crops. Climatic conditions, soil properties, and crop type that control microclimates can affect the hydrolysis of applied fertilizers and their responses to mitigation options (Peng et al., 2006; Abdo et al., 2020). Urea hydrolysis under the wheat system with wet-dry cycles is higher than other mineral N fertilizers, while waterlogged conditions of rice resulted in higher hydrolysis of OCF (i.e., ammonium bicarbonate and diammonium phosphate) than urea (Zhang et al., 2011). Replacing urea with other N sources including OA and IU often reduced AP, GWP, and AEP but was restricted by the reductions in yield owing to the high readily N content of urea and almost studies used lower N rates of IU than urea (Abdo et al., 2020). Mulch and biochar caused yield increases and AEP reductions but increased AP and GWP. Mulch and biochar increase gas emissions by raising soil C:N ratio and pH in addition to improving crop yield by augmenting the retention of ${\text{NO}}_{3}^{-}$-N and ${\text{NH}}_{4}^{+}$-N against leaching (Sun et al., 2018; Tian et al., 2020).

## Study limitations and uncertainties

The current study included a wide range of comparisons about mitigation strategies of environmental hazards induced by Nr losses, thus almost available data were collected. Few comparisons including biochar and inhibitors effect on AEP of wheat, maize, and rice, OCF on AEP of wheat and maize, mulch and R1 on rice AEP, and IU on rice GWP could not be set using meta-analysis due to a shortage of datasets. These comparisons were rejected by the meta-analysis due to a low number of pairwise comparisons which caused a higher heterogeneity p-value than 0.05. Almost studies attributed Nr losses to the cultivated area; however, the current study used the yield-scaled values to reflect the dual goals of sustainable intensification about achieving higher yields while reducing environmental hazards (Zhou and Butterbach-Bahl, 2014). Ammonia volatilization and nitrate leaching are indirect sources of nitrous oxide emissions, but they were not included in the calculations of GWP as only 0.75–1% of them are lost as N_2_O (IPPC, 2006). Nitrous oxide could contribute to the acidification potential by 31% (Brentrup et al., 2004), but it was neglected in the calculations of AEP as N_2_O is relatively stable in air for 114 years. Besides, the main pathway for diffuse Nr losses to aquatic ecosystems from soils is via ${\text{NO}}_{3}^{-}$ leaching (Brentrup et al., 2004). The equivalency factors were used to calculate AP, GWP, and AEP since there are no direct measurements. All previous works have used these converters when assessing the environmental impacts of agricultural production systems (Brentrup et al., 2004; Chen et al., 2014).

## Conclusion

The current study calculated great amounts of AP, GWP, and AEP (ranging between 17 and 33 Tg, 7 and 10 Tg, and 6 and 87 Gg, respectively) under the conventional N fertilization to produce 131.4, 257.2, and 212.1 Tg of wheat, maize, and rice, respectively. For its importance in achieving equiponderant yield production and environmental impact, sustainable agricultural intensification has been intensively studied using different strategies. Our study refuted the strategy of reducing the N rate due to the great reductions in yield, although it achieved a great decrease in AP, GWP, and AEP. More restrictions should be set against the increase in N rate over the present conventional rates till developing more effective strategies. Inhibitors usage and subsurface application is a promising strategy that produced more grains while reduced AP, GWP, and AEP significantly in wheat. Additionally, inhibitor usage and biochar are effective strategies that could achieve the sustainable intensification of maize production. None of the investigated strategies reduced all the environmental impacts of rice production while optimizing yield, thus new technologies should be developed.

## Data availability statement

The original contributions presented in the study are included in the article/Supplementary material, further inquiries can be directed to the corresponding authors.

## Author contributions

AA and EA-H: conceptualization. AA, JY, and DS: data curation. AA and YL: formal analysis. JZ: funding acquisition. AA and HW: investigation. AA and MM: methodology and visualization. HW and JZ: project administration, resources, validation, and writing—review and editing. AA and DS: software. JZ: supervision. AA, JY, and EA-H: writing—original draft. All authors contributed to the article and approved the submitted version.

## Funding

This study was supported by the Science and Technology Planning Project of Guangdong Province of China (Grant Nos. 2019B030301007 and 2021B0202030002), Laboratory of Lingnan Modern Agriculture Project (NT2021010), and Guangdong Provincial Special Project of Rural Revitalization Strategy (Document No. [2021] 12).

## Conflict of interest

The authors declare that the research was conducted in the absence of any commercial or financial relationships that could be construed as a potential conflict of interest.

## Publisher's note

All claims expressed in this article are solely those of the authors and do not necessarily represent those of their affiliated organizations, or those of the publisher, the editors and the reviewers. Any product that may be evaluated in this article, or claim that may be made by its manufacturer, is not guaranteed or endorsed by the publisher.
