# Supplementary material for: Coupling the environmental impacts of reactive nitrogen losses and yield responses of staple crops in China
Source: Front Plant Sci. 2022 Aug 19;13:927935. doi: 10.3389/fpls.2022.927935 (PMC9450997; doi:10.3389/fpls.2022.927935)
Supplement: Supplementary file 1 [file Table_1.DOCX]

**Table S1.** Total grain yield in each province and China (Tg) and the accompanied acidification potential (AP) (Tg Acid equiv.), global warming potential (GWP) (Tg CO_2_ equiv.) and aquatic eutrophication potential (AEP) (Gg PO_4_ equiv.).

| **Province** | **Grain yield (Tg)** | | | **AP (Tg Acid equiv.)** | | | **GWP (Tg CO_2_ equiv.)** | | | **AEP (Gg PO_4_ equiv.)** | | |
| --- | --- | --- | --- | --- | --- | --- | --- | --- | --- | --- | --- | --- |
| **Beijing** | 0.05 | 0.27 | 0.00 | 0.01 | 0.03 | 0.00 | 0.00 | 0.01 | 0.00 | 0.02 | 0.09 | 0.00 |
| **Tianjin** | 0.57 | 1.11 | 0.37 | 0.08 | 0.12 | 0.06 | 0.04 | 0.04 | 0.01 | 0.21 | 0.38 | 0.01 |
| **Hebei** | 14.51 | 19.41 | 0.53 | 1.91 | 2.11 | 0.08 | 1.08 | 0.63 | 0.02 | 5.37 | 6.60 | 0.02 |
| **Shanxi** | 2.29 | 9.82 | 0.01 | 0.30 | 1.07 | 0.00 | 0.17 | 0.32 | 0.00 | 0.85 | 3.34 | 0.00 |
| **Inner Mongolia** | 2.02 | 27.00 | 1.22 | 0.27 | 2.93 | 0.19 | 0.15 | 0.88 | 0.04 | 0.75 | 9.18 | 0.04 |
| **Liaoning** | 0.01 | 16.63 | 4.18 | 0.00 | 1.81 | 0.64 | 0.00 | 0.54 | 0.15 | 0.01 | 5.65 | 0.13 |
| **Jilin** | 0.00 | 28.00 | 6.46 | 0.00 | 3.04 | 0.99 | 0.00 | 0.91 | 0.23 | 0.00 | 9.52 | 0.19 |
| **Heilongjiang** | 0.36 | 39.82 | 26.86 | 0.05 | 4.33 | 4.10 | 0.03 | 1.30 | 0.94 | 0.13 | 13.54 | 0.81 |
| **Shanghai** | 0.13 | 0.01 | 0.88 | 0.02 | 0.00 | 0.13 | 0.01 | 0.00 | 0.03 | 0.05 | 0.00 | 0.03 |
| **Jiangsu** | 12.89 | 3.00 | 19.58 | 1.69 | 0.33 | 2.99 | 0.96 | 0.10 | 0.69 | 4.77 | 1.02 | 0.59 |
| **Zhejiang** | 0.36 | 0.21 | 4.77 | 0.05 | 0.02 | 0.73 | 0.03 | 0.01 | 0.17 | 0.13 | 0.07 | 0.14 |
| **Anhui** | 16.08 | 5.96 | 16.81 | 2.11 | 0.65 | 2.57 | 1.20 | 0.19 | 0.59 | 5.95 | 2.03 | 0.50 |
| **Fujian** | 0.00 | 0.13 | 3.98 | 0.00 | 0.01 | 0.61 | 0.00 | 0.00 | 0.14 | 0.00 | 0.04 | 0.12 |
| **Jiangxi** | 0.03 | 0.16 | 20.92 | 0.00 | 0.02 | 3.20 | 0.00 | 0.01 | 0.74 | 0.01 | 0.05 | 0.63 |
| **Shandong** | 24.72 | 26.07 | 0.99 | 3.25 | 2.83 | 0.15 | 1.84 | 0.85 | 0.03 | 9.15 | 8.86 | 0.03 |
| **Henan** | 36.03 | 23.51 | 5.01 | 4.74 | 2.55 | 0.77 | 2.68 | 0.77 | 0.18 | 13.33 | 7.99 | 0.15 |
| **Hubei** | 4.10 | 3.23 | 19.66 | 0.54 | 0.35 | 3.00 | 0.31 | 0.11 | 0.69 | 1.52 | 1.10 | 0.59 |
| **Hunan** | 0.08 | 2.03 | 26.74 | 0.01 | 0.22 | 4.09 | 0.01 | 0.07 | 0.94 | 0.03 | 0.69 | 0.80 |
| **Guangdong** | 0.00 | 0.55 | 10.32 | 0.00 | 0.06 | 1.58 | 0.00 | 0.02 | 0.36 | 0.00 | 0.19 | 0.31 |
| **Guangxi** | 0.01 | 2.73 | 10.16 | 0.00 | 0.30 | 1.55 | 0.00 | 0.09 | 0.36 | 0.00 | 0.93 | 0.30 |
| **Hainan** | 0.00 | 0.00 | 1.31 | 0.00 | 0.00 | 0.20 | 0.00 | 0.00 | 0.05 | 0.00 | 0.00 | 0.04 |
| **Chongqing** | 0.08 | 2.51 | 4.87 | 0.01 | 0.27 | 0.74 | 0.01 | 0.08 | 0.17 | 0.03 | 0.85 | 0.15 |
| **Sichuan** | 2.47 | 10.66 | 14.79 | 0.33 | 1.16 | 2.26 | 0.18 | 0.35 | 0.52 | 0.92 | 3.63 | 0.44 |
| **Guizhou** | 0.33 | 2.59 | 4.21 | 0.04 | 0.28 | 0.64 | 0.02 | 0.08 | 0.15 | 0.12 | 0.88 | 0.13 |
| **Yunnan** | 0.74 | 9.26 | 5.28 | 0.10 | 1.01 | 0.81 | 0.06 | 0.30 | 0.19 | 0.27 | 3.15 | 0.16 |
| **Tibet** | 0.20 | 0.03 | 0.01 | 0.03 | 0.00 | 0.00 | 0.01 | 0.00 | 0.00 | 0.07 | 0.01 | 0.00 |
| **Shaanxi** | 4.01 | 5.84 | 0.81 | 0.53 | 0.63 | 0.12 | 0.30 | 0.19 | 0.03 | 1.48 | 1.99 | 0.02 |
| **Gansu** | 2.81 | 5.90 | 0.03 | 0.37 | 0.64 | 0.00 | 0.21 | 0.19 | 0.00 | 1.04 | 2.01 | 0.00 |
| **Qinghai** | 0.43 | 0.12 | 0.00 | 0.06 | 0.01 | 0.00 | 0.03 | 0.00 | 0.00 | 0.16 | 0.04 | 0.00 |
| **Ningxia** | 0.42 | 2.35 | 0.67 | 0.05 | 0.25 | 0.10 | 0.03 | 0.08 | 0.02 | 0.15 | 0.80 | 0.02 |
| **Xinjiang** | 5.72 | 8.28 | 0.73 | 0.75 | 0.90 | 0.11 | 0.43 | 0.27 | 0.03 | 2.12 | 2.81 | 0.02 |
| **China** | 131.44 | 257.18 | 212.13 | 17.28 | 27.94 | 32.42 | 9.78 | 8.37 | 7.45 | 48.63 | 87.44 | 6.36 |


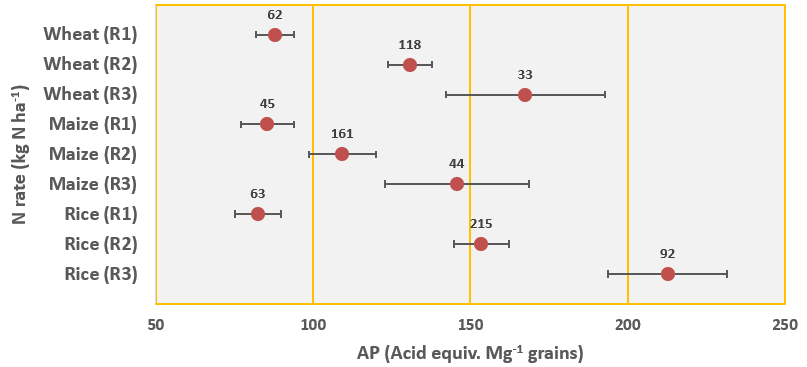


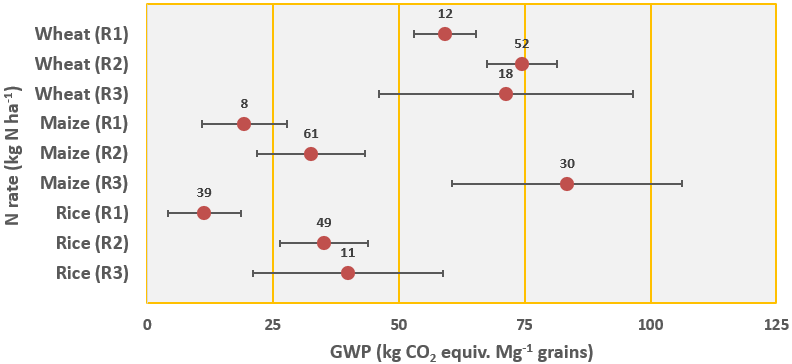


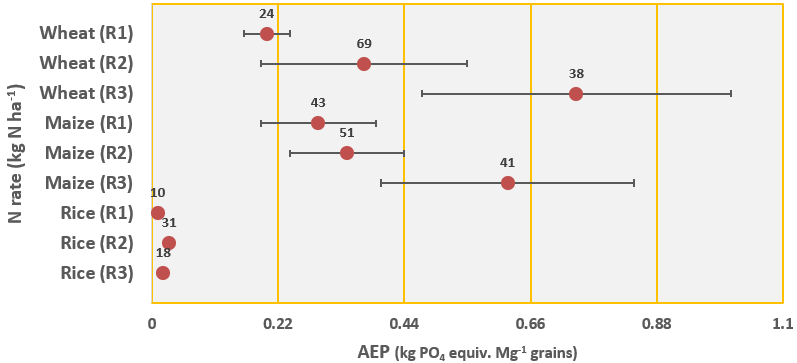


**Fig S1 (A):** The responses of AP, GWP and AEP to the reduced rate strategy in the three crops (wheat, maize and rice). AP is acidification potential (Acid equiv. Mg^-1^ grains), GWP is global warming potential (kg CO_2_ equiv. Mg^-1^ grains) and AEP is aquatic eutrophication potential (kg PO_4_ equiv. Mg^-1^ grains). R1 is the reduced rate strategy, R2 is the conventional rate used in China (between 150-250, 200-260 and 170-260 kg N ha^-1^ for wheat, maize and rice, respectively) and R3 is the increased rate scenario. Numbers above the markers are the number of observations.


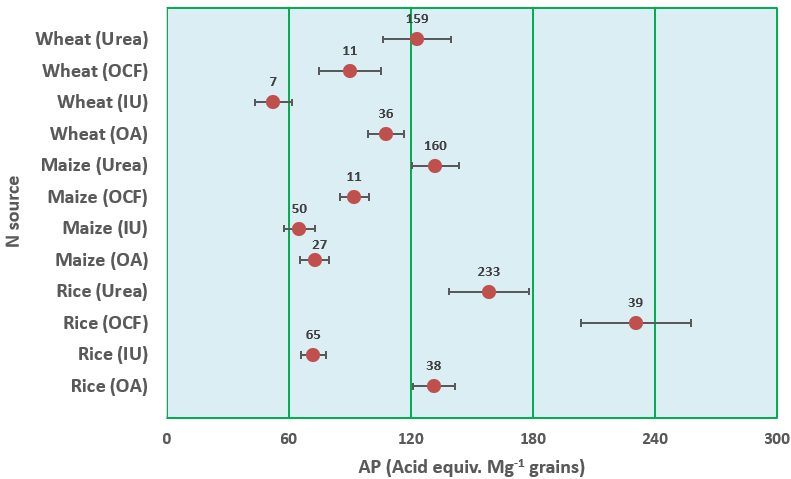


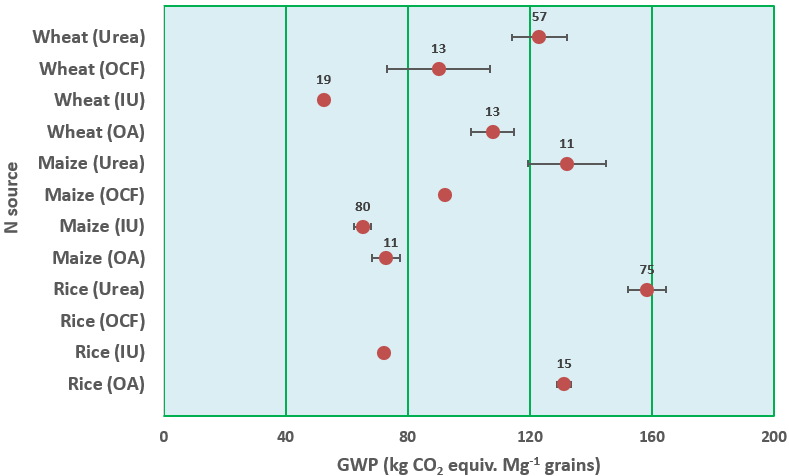


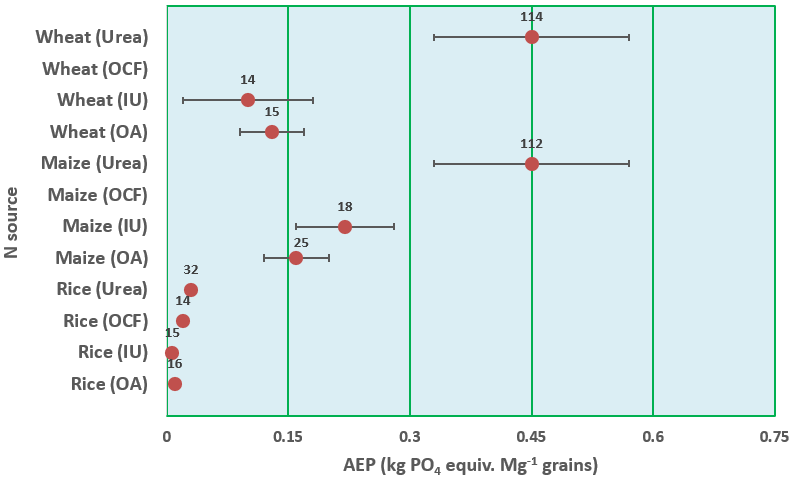


**Fig S1 (B):** The responses of AP, GWP and AEP to the nitrogen (N) source strategy in the three crops (wheat, maize and rice). AP is acidification potential (Acid equiv. Mg^-1^ grains), GWP is global warming potential (kg CO_2_ equiv. Mg^-1^ grains) and AEP is aquatic eutrophication potential (kg PO_4_ equiv. Mg^-1^ grains). These N sources are urea, other synthetic fertilizers (OCF), improved urea (IU, slow released fertilizers) and organic sources (OA). Numbers above the markers are the number of observations.


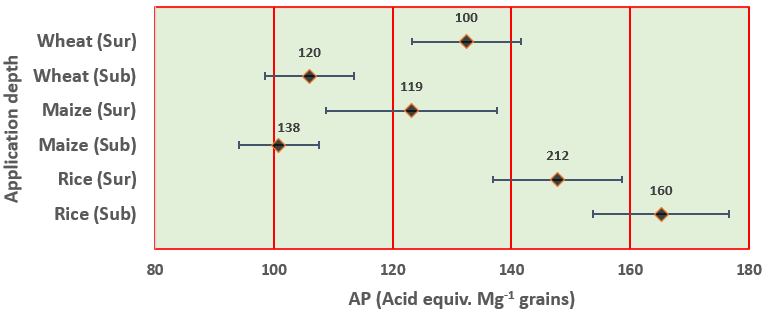


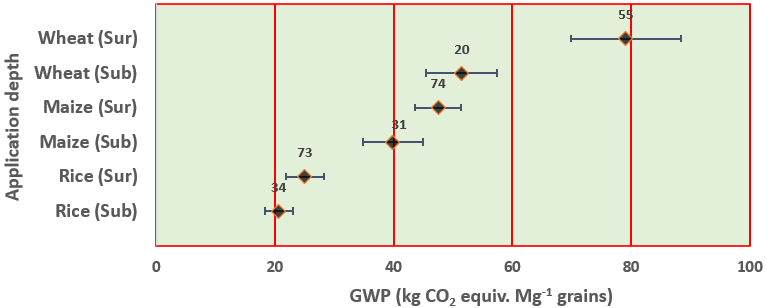


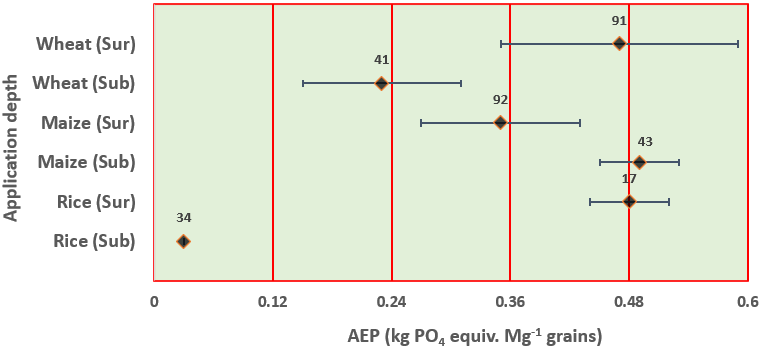


**Fig S1 (C):** The responses of AP, GWP and AEP to the application depth strategy in the three crops (wheat, maize and rice). AP is acidification potential (Acid equiv. Mg^-1^ grains), GWP is global warming potential (kg CO_2_ equiv. Mg^-1^ grains) and AEP is aquatic eutrophication potential (kg PO_4_ equiv. Mg^-1^ grains). Sur is the surface application and Sub is the subsurface application. Numbers above the markers are the number of observations.


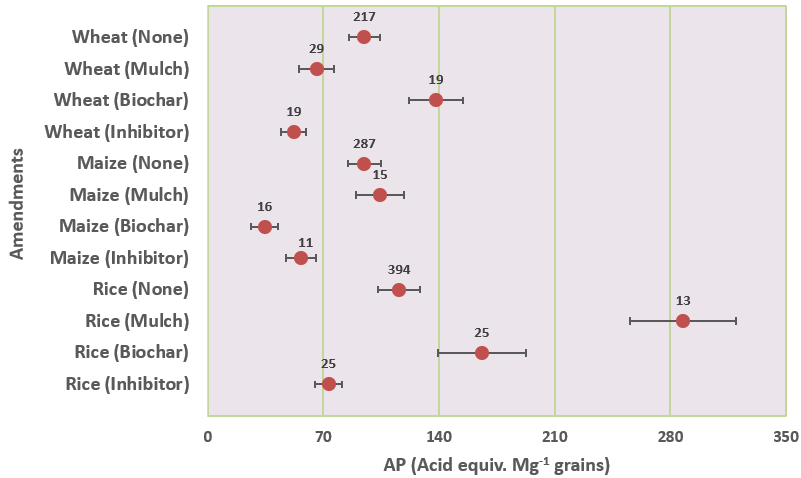


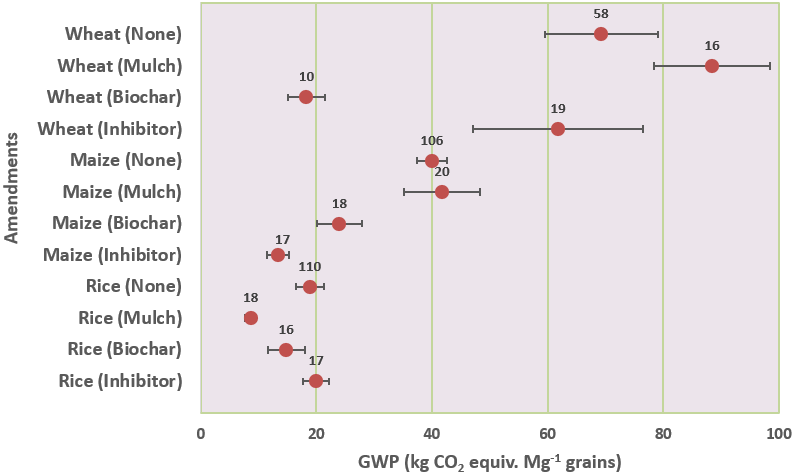


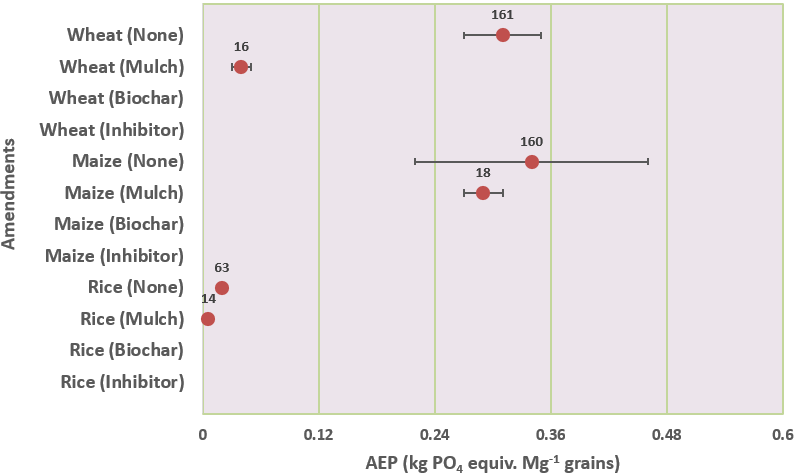


**Fig S1 (D):** The responses of AP, GWP and AEP to the application depth strategy in the three crops (wheat, maize and rice). AP is acidification potential (Acid equiv. Mg^-1^ grains), GWP is global warming potential (kg CO_2_ equiv. Mg^-1^ grains) and AEP is aquatic eutrophication potential (kg PO_4_ equiv. Mg^-1^ grains). None means no amendments were applied. Numbers above the markers are the number of observations.
